# Analogy between Gastritis and Hydrophobia

**Published:** 1813-06

**Authors:** C. Aber

**Affiliations:** Halesworth


*To the Editors of the Medical and Physical Journal.*

GENTLEMEN,

ALL the friends of humanity must exult at the triumph of the healing art over that most horrible, and hitherto most refractory of all diseases, Hydrophobia; and all candid men must be anxious to pay due honor to every one, who, by reasoning on the statement of facts, has accelerated this most splendid and most happy event.

Induced by these reflections, I beg leave, through the medium of your highly-valuable publication, to call the attention of Dr. Kinglake to the communication of Mr. Borrett, of Yarmouth, in the 122d Number of the Medical and Physical Journal, in which he will find himself anticipated in his observations on the analogy existing between Gastritis and Hydrophobia, as well as in his practical conclusions. Dr. Kinglake will also observe, that Mr. Borrett's paper was the more valuable, as giving the result of a very close investigation of the symptoms of an unequivocal instance of the disease, and of the morbid appearances discovered on dissection.

In this appeal to Dr. Kinglake, I am only desirous of doing justice to Mr. Borrett, and of bringing to the notice of the former gentleman a paper which elucidates his own ingenious reasoning, and confirms the accuracy of his induction. I am, Gentlemen,

Your obedient and respectful Servant,

*Halesworth, April* 19, 1813. C. ABER.

---

*To the Editors of the Medical and Physical Journal.*

